# Enhancing Patient Safety in Refractory Ventricular Fibrillation: A Systematic Review of Double Sequential and Vector Change Defibrillation Barriers

**DOI:** 10.3390/healthcare13202645

**Published:** 2025-10-21

**Authors:** Kyriakos Alexandrou, Elina Khattab, Evanthia Asimakopoulou

**Affiliations:** 1 Department of Nursing, School of Health Sciences, Frederick University, 1036 Nicosia, Cyprus; hsc.ae@frederick.ac.cy; 2 Cardiology Department, Nicosia General Hospital, 2029 Nicosia, Cyprus; khattab_elina@outlook.com

**Keywords:** ventricular fibrillation, defibrillators, electric countershock, cardiac arrest, resuscitation, treatment outcome, neurologic manifestations

## Abstract

**Background/Objectives:** Ventricular fibrillation (VF) is the most common shockable rhythm in cardiac arrest, yet refractory VF (RVF), defined as persistent VF after ≥three failed defibrillation attempts, poses a significant challenge. Two alternative strategies, double sequential external defibrillation (DSED) and vector change (VC) defibrillation, aim to enhance defibrillation success where conventional methods fail. This review evaluates the clinical feasibility, safety, and implementation barriers of DSED and VC in RVF cases. **Methods:** A systematic review was conducted following PRISMA 2020 guidelines. PubMed, Scopus, and CINAHL databases were searched for studies published between January 2015 and August 2025. Eligible studies included adult RVF patients treated with DSED or VC. Outcomes assessed included implementation barriers, safety concerns, and methodological limitations. Study quality was evaluated using the Newcastle–Ottawa Scale and the Cochrane RoB 2 tool. **Results:** Sixteen studies met the inclusion criteria. Identified barriers were grouped into practical and methodological categories. Practical barriers included the need for dual defibrillators and pads, delays in shock coordination, inconsistent protocols, equipment compatibility issues, and dependence on trained personnel. Methodological barriers included small sample sizes, retrospective designs, inconsistent RVF definitions, and incomplete reporting of neurological outcomes. **Conclusions:** DSED and VC defibrillation may offer potential benefits in managing RVF, but their use is hindered by significant practical and methodological barriers. Due to the limited number of randomized trials, further high-quality studies with standardized definitions and safety endpoints are needed to clarify their clinical utility and inform implementation.

## 1. Introduction

Cardiac arrest remains a leading cause of sudden death globally, with ventricular fibrillation (VF) being the most common shockable rhythm encountered [1]. It is categorized into out-of-hospital (OHCA) and in-hospital cardiac arrest (IHCA), each with distinct epidemiological patterns and management challenges [2]. Despite advancements in cardiopulmonary resuscitation (CPR) and defibrillation protocols, a significant proportion of patients develop refractory ventricular fibrillation (RVF)—defined as VF persisting after at least three failed shocks [2,3]. This condition necessitates exploring alternative defibrillation strategies to improve outcomes without compromising patient safety, particularly in high-acuity settings [1].

According to the latest guidelines from the European Resuscitation Council (ERC) and the American Heart Association (AHA), single-shock defibrillation is recommended for shockable rhythms, including VF and pulseless ventricular tachycardia [4,5]. However, neither organization currently provides specific guidance on alternative methods like double sequential or vector change defibrillation, despite their potential relevance in refractory VF [6]. The only related mention is in the broader context of refractory cardiac arrest, where eCPR may be considered under certain conditions [4,5].

Among the proposed strategies, double sequential external defibrillation (DSED) and vector change (VC) defibrillation are gaining attention. DSED administers two rapid, sequential shocks from separate defibrillators, while VC alters pad placement to change current direction through the myocardium [7,8,9]. Preliminary evidence from observational studies and small randomized trials suggests both may increase return of spontaneous circulation (ROSC), especially in OHCA [10,11].

Despite early investigations, widespread use of DSED and VC is limited. Practical barriers such as the need for dual defibrillators, coordination delays, lack of standardized protocols, and dependence on adequately trained personnel may compromise timely defibrillation and safety [3,12]. Methodological barriers, including small sample sizes, heterogeneous study designs, inconsistent RVF definitions, and incomplete outcome reporting, further limit the generalizability of current evidence [13,14,15].

Given these issues, the effectiveness and safety of DSED and VC remain debated. This systematic review synthesizes current evidence on their use in RVF and critically evaluates the practical and methodological barriers to their implementation. For clarity, practical barriers refer to real-world factors affecting clinical application, while methodological barriers concern limitations in study design and reporting. Emphasis is placed on the implications for patient safety and the need to inform future clinical practice.

## 2. Materials and Methods

This systematic review was conducted in accordance with the Preferred Reporting Items for Systematic Reviews and Meta-Analyses (PRISMA 2020) guidelines [16]. The review protocol specified the objectives, eligibility criteria, search strategy, and data analysis plan, and was registered in the PROSPERO database (registration number: 1144647). The completed PRISMA 2020 checklist is available as Supplementary File S1.

### 2.1. Search Strategy

A comprehensive literature search was performed in the PubMed, Scopus, and CINAHL databases, covering the period from January 2015 to August 2025 (last updated on 15 August 2025). The search strategy combined keywords and Medical Subject Headings (MeSH), including: “practical barriers,” “neurological outcomes,” “survival,” “return of spontaneous circulation,” “defibrillation strategies,” “cardiac arrest,” “refractory ventricular fibrillation,” and “double sequential defibrillation.” Boolean operators (“AND,” “OR”) were used to refine the search. The search was limited to English-language, full-text publications involving human subjects.

### 2.2. PICOT Framework 

The review question was structured using the PICOT framework to ensure methodological clarity and to align with clinical research standards:

- **P** (Population): Adult patients (≥18 years) who experienced refractory ventricular fibrillation (RVF) during cardiac arrest.
- **I** (Intervention): Double sequential external defibrillation (DSED) or vector change (VC) defibrillation.
- **C** (Comparison): Conventional single-shock defibrillation or standard resuscitation protocol.
- **O** (Outcomes): Return of spontaneous circulation (ROSC), survival to hospital discharge, neurological outcomes, and identification of practical barriers, especially those affecting patient safety.
- **T** (Time): Studies published between January 2015 and August 2025.

### 2.3. Eligibility Criteria

Studies were included if they were primary clinical investigations, such as randomized controlled trials (RCTs), prospective or retrospective cohort studies, or case series involving adult patients (aged ≥ 18 years) who experienced RVF during cardiac arrest. Eligible studies assessed the use of DSED or VC defibrillation and reported at least one of the following outcomes: return of spontaneous circulation (ROSC), survival to hospital discharge, neurological outcomes, or barriers to implementation, with a particular focus on patient safety.

Exclusion criteria were: narrative reviews, other systematic reviews, meta-analyses, editorials, commentaries, and conference abstracts lacking original data. Studies without methodological clarity or extractable outcomes were also excluded. Only English-language articles with accessible full texts were considered. Secondary analyses of primary trial data were included only if they presented novel findings relevant to the review objectives.

### 2.4. Study Selection

**Figure 1:**
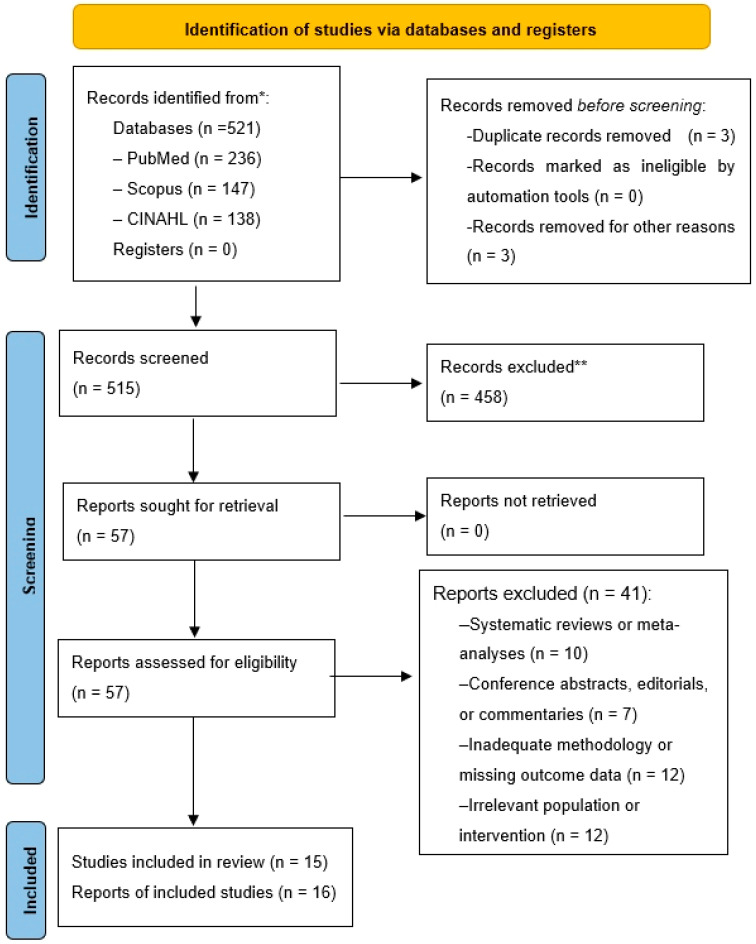
PRISMA 2020 flow diagram of the study selection process. * Consider, if feasible to do so, reporting the number of records identified from each database or register searched (rather than the total number across all databases/registers). ** If automation tools were used, indicate how many records were excluded by a human and how many were excluded by automation tools. Source: Page MJ et al. BMJ 2021;372:n71. doi: 10.1136/bmj.n71 [17]. This work is licensed under CC BY 4.0. To view a copy of this license, visit https://creativecommons.org/licenses/by/4.0/ (accessed on 18 October 2025).

Two independent reviewers screened titles and abstracts of all records retrieved. Full-text articles were then assessed based on predefined inclusion and exclusion criteria. Disagreements were resolved through discussion and consensus. The selection process is illustrated in the PRISMA 2020 flow diagram (Figure 1).

### 2.5. Data Extraction and Management

Data were extracted independently by two reviewers using a standardized extraction form. Extracted variables included: first author, publication year, study design, sample size, intervention characteristics (DSED or VC), and primary outcomes (e.g., ROSC, survival to discharge, neurological status, and practical barriers). Special focus was placed on patient safety outcomes, including delays in defibrillation, procedural complications, and risks associated with implementation in emergency or high-acuity settings.

### 2.6. Use of Generative AI

No generative artificial intelligence tools were used in the design, conduct, data extraction, or analysis of this review. Minor language refinements were made using conventional word processing software.

## 3. Results

### 3.1. Study Characteristics 

Two RCTs were included in this review: one pilot cluster-randomized trial with crossover design [11] and one full cluster-randomized controlled trial [18]. Most of the remaining studies were retrospective or observational in design [1,9,10,13,14,16,17,18,19,20,21,22,23,24]. Additionally, one study was a secondary analysis of a cluster-randomized trial [24], and one was an observational study based on registry data from a previously conducted cluster trial [15].

Neurological outcomes were reported inconsistently, with six studies including some measure of neurological status [9,16,17,18,19,23,24]. ROSC and survival to hospital discharge were reported in the majority of studies, specifically in twelve out of sixteen [1,9,13,14,16,17,18,19,20,22]. Methodological limitations were also common, with eleven studies using retrospective designs [1,9,13,14,16,17,18,19,20,21,22,24], five with small sample sizes [1,9,13,16,24], and four noting a lack of standardized implementation procedures [19,21,23,25]. A detailed overview of study characteristics is provided in Table 1.

### 3.2. Quality Assessment

The methodological quality of included studies was evaluated using standardized tools: the Newcastle–Ottawa Scale (NOS) for observational studies and the RoB 2 (Revised Cochrane Risk of Bias) tool for RCTs. Case series were assessed using the Joanna Briggs Institute (JBI) Critical Appraisal Checklist for Case Series.

#### 3.2.1. Newcastle–Ottawa Scale

The results of the NOS assessment for observational studies are presented in Table 2.

**Figure 2:**
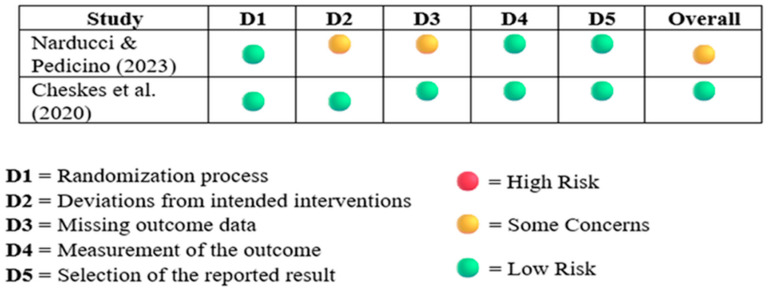
Risk of bias assessment of RCT using the Cochrane RoB 2 tool. Domains: D1 = Randomization process; D2 = Deviations from intended interventions; D3 = Missing outcome data; D4 = Measurement of the outcome; D5 = Selection of the reported result. Color coding: Green = Low risk; Yellow = Some concerns; Red = High risk. Adapted from Cheskes et al. (2020) [11] and Narducci & Pedicino (2023) [18].

The methodological quality of the included observational studies was assessed using the Newcastle–Ottawa Scale (NOS). As shown in Figure 2, the total NOS scores ranged from 6 to 8 stars out of a maximum of 9, indicating a generally moderate to high quality among the selected studies. Most studies demonstrated appropriate representativeness and ascertainment of exposure, while a few lacked clear follow-up adequacy or had limitations in comparability due to insufficient adjustment for confounders. Notably, only one study achieved the maximum score, and only two studies scored below 7, suggesting overall acceptable methodological robustness for inclusion in this review.

#### 3.2.2. Revised Cochrane Risk of Bias Tool for RCT 2

The risk of bias of RCT was assessed using the RoB 2 tool, and the results are illustrated in Figure 2.

The risk of bias assessment using the Cochrane RoB 2 tool revealed that one RCT was judged as having low risk of bias across all domains, while the second study had some concerns in domains related to deviations from intended interventions and missing outcome data. Overall, both studies demonstrated acceptable methodological quality, though minor limitations in adherence and data completeness should be considered when interpreting their findings.

#### 3.2.3. The Joanna Briggs Institute Critical Appraisal Checklist for Case Series

Case series were assessed using the JBI Critical Appraisal Checklist for Case Series, which consists of 10 questions evaluating study inclusion criteria, reliability of case identification, reporting of demographics and outcomes, and appropriateness of analysis. Responses were scored as Yes, No, Unclear, or Not Applicable (NA).

The results of the JBI Critical Appraisal Checklist for Case Series are presented in Table 3 below.

This table presents the quality assessment of the primary studies based on ten criteria (Q1–Q10). Each criterion corresponds to a specific question addressing study aims, clarity of the research question, population description, methodological rigor, reliability of results, and risk of bias. Responses are categorized as “Yes” (criterion fully met), “No” (criterion not met), or “Unclear” (insufficient information reported).

The methodological quality assessment of the included case series demonstrated generally strong performance across most JBI Checklist domains, with explicit inclusion criteria, reliable condition measurement, valid identification methods, and comprehensive case inclusion and follow-up. The only consistent limitations were found in the reporting of demographics and, in some cases, clinical information, as well as the lack of appropriate statistical analysis. Despite the overall high reporting standards, the absence of statistical analyses reduces the potential for robust evidence synthesis. Thus, these case series are methodologically sound but would benefit from enhanced demographic/clinical reporting and incorporation of appropriate statistical methods to strengthen their scientific contribution. The full detailed checklist along with specific comments and clarifications are provided in Supplementary File S2.

### 3.3. Practical Barriers and Patient Safety Implications in the Implementation of DSED and VC Techniques

Understanding the real-world feasibility of advanced defibrillation strategies is essential to improving outcomes in shock-RVF. Both DSED and VC have been proposed as alternative techniques following failed standard shocks, with evidence suggesting higher termination and ROSC rates in certain contexts [11,18]. This review aimed to identify and synthesize the practical barriers to the implementation of these strategies in OHCA. The analysis revealed four key categories of recurring challenges. These barriers are primarily practical but also have direct implications for patient safety, particularly when delays, complexity, or equipment constraints impede timely defibrillation during critical resuscitation windows [11,18].

#### 3.3.1. Equipment and Resource Limitations

The requirement for two defibrillators and a second set of electrode pads represents a persistent operational challenge for DSED use, particularly in prehospital settings with limited equipment availability [13,18,25]. Additionally, the DSED setup has been associated with delays in shock delivery, with one study reporting a mean delay of approximately 13.7 s compared to standard defibrillation [10]. These constraints may delay life-saving interventions and increase the likelihood of poor outcomes, particularly in resource-limited or rural EMS environments where immediate access to two defibrillators is not guaranteed [18,24].

#### 3.3.2. Coordination and Timing Complexity

The clinical efficacy of DSED is likely influenced by the inter-shock interval. Shorter intervals (<75 ms) were associated with significantly improved VF termination [14], but achieving near-simultaneous shocks in the field requires precise coordination between responders. Furthermore, concerns about potential defibrillator damage during closely timed shocks have limited uptake in some EMS systems [12,14]. Inadequate coordination or misfiring due to device incompatibility may lead to ineffective shocks or further deterioration, raising safety concerns in high-stakes clinical settings [14].

### 3.4. Training and Personnel Constraints

DSED application often depends on the presence of advanced or specially trained personnel, particularly in EMS environments [11,22]. Managing pad placement, ensuring vector configuration, and synchronizing two devices add considerable operational complexity, especially in high-stress scenarios [9,20]. The dependence on experienced personnel and precise execution increases the risk of procedural errors, which in turn may compromise the safety of patients requiring rapid, standardized interventions [22].

### 3.5. Protocol Inconsistency and Limited Integration

Variation in DSED and VC protocols across systems introduces additional barriers. Some EMS agencies initiate DSED after three shocks, while others wait for five or more [18,22]. Differences also exist in energy delivery and pad configuration. Moreover, in several settings, medical control authorization is required before DSED can be administered, contributing to treatment delays [21]. Although VC can be deployed with a single defibrillator by repositioning pads, it may provide lower clinical benefit than DSED [18,25]. The lack of unified protocols and delayed authorization requirements may contribute to treatment variability and delayed defibrillation, jeopardizing time-sensitive outcomes and patient safety.

### 3.6. Limitations of the Evidence

Although recent studies investigating DSED and VC offer important insights into their feasibility and potential benefits in OHCA, several methodological and evidentiary limitations undermine the robustness and limit the generalizability of current findings. Many of the methodological limitations identified also have implications for safety assessments, making it difficult to evaluate whether the procedures were not only effective but also safe under real-world conditions. These limitations can be grouped into four primary domains:

#### 3.6.1. Small Sample Sizes and Underpowered Designs

The majority of included studies involved limited patient cohorts, with several relying on pilot data or small case series [1,9,24]. Even larger trials, such as Narducci and Pedicino [18], were statistically underpowered due to early termination and enrolled fewer participants than initially planned, reducing the confidence in effect estimates.

#### 3.6.2. Retrospective and Observational Methodologies

Most studies employed retrospective registry reviews or observational designs, lacking randomization or blinding [13,20,21]. This introduces risk for selection bias, residual confounding, and variability in protocol implementation, particularly regarding the timing of DSED application, pad placement, and CPR quality [19,23].

#### 3.6.3. Lack of Standardized Outcomes and Comparator Arms

Several studies did not include control groups or failed to report critical clinical endpoints such as neurological recovery or post-resuscitation interventions [15,22]. Others were constrained by incomplete datasets [11] or by unclear comparator definitions and inconsistent treatment protocols [11,16].

#### 3.6.4. Simulation-Based and Non-Generalizable Settings

At least one study utilized only simulation data [10], which, while informative for logistical planning, cannot replicate the physiologic and operational complexity of real-life cardiac arrests. Moreover, several trials were conducted in urban EMS environments with high resource availability, limiting external validity in lower-resource or rural settings [11,25].

In summary, although DSED and VC demonstrate procedural feasibility and promise in improving resuscitation outcomes, the current evidence base remains constrained by methodological shortcomings, including small samples, retrospective designs, limited outcome reporting, and lack of protocol consistency. These gaps highlight the need for well-powered, multicenter RCTs to guide future practice and policy.

### 3.7. Comparative Effectiveness of DSED, VC, and Conventional Defibrillation

Randomized evidence consistently favors DSED over conventional defibrillation, showing higher rates of ROSC, survival to discharge, and better neurological outcomes [18,25]. In the DOSE-VF trial, survival reached 30% with DSED, versus 13% with standard shocks, while VC achieved intermediate outcomes. Although VF termination was highest with VC, the survival benefit was more evident with DSED [11]. However, only six studies explicitly reported neurological outcomes, limiting the strength of conclusions regarding post-arrest neurological recovery [9,16,17,18,19,23,24].

Observational studies report mixed findings, with some cohorts showing no clear advantage or even lower ROSC with DSED [19,20]. Nonetheless, registry data suggest early application of DSED may improve ROSC over prolonged standard defibrillation [21].

In summary, randomized trials support DSED as the most effective approach for survival and neurological outcomes. VC appears beneficial for VF termination, but has not shown a consistent survival advantage. Observational results remain heterogeneous, influenced by protocol variation and timing of intervention. While DSED shows promise in improving survival and neurological outcomes, further research is needed to confirm that its implementation does not introduce unintended safety risks, especially in prehospital or resource-limited environments. While no pooled statistical analysis was performed, reported ROSC rates across the included studies varied between 24% and 46%, with an estimated central tendency around 31%, highlighting both variability in implementation and heterogeneity in study design [1,9,13,14,16,18,22].

## 4. Discussion 

This systematic review highlights a dual challenge in the implementation of DSED and VC for RVF. On one hand, significant clinical and practical barriers limit the feasibility of these strategies in real-world practice. On the other hand, the existing evidence base is methodologically constrained, with small sample sizes, inconsistent designs, and incomplete reporting, leading to considerable uncertainty in interpreting outcomes. Together, these issues underscore both the promise and the fragility of current findings. Importantly, these challenges have implications not only for effectiveness but also for patient safety, as delays or misapplication in critical moments may reduce the likelihood of favorable neurological outcomes.

One of the most consistent barriers is the need for two defibrillators and an additional set of pads [13,18,25]. While this requirement may be manageable in well-resourced urban EMS systems, it poses a significant obstacle in most prehospital environments where even a single defibrillator may not be available. In many countries, limited budgets, the cost of disposable pads, and the absence of widespread deployment of advanced defibrillators create a practical barrier. Notably, even within hospitals, DSED may not always be feasible, particularly in resource-constrained settings or smaller facilities. These practical constraints likely explain why adoption has been limited outside of specialized centers. When such equipment is unavailable or inconsistently applied, patient safety may be compromised due to delayed or suboptimal interventions during time-critical resuscitation efforts.

Delays in shock delivery also represent a practical barrier. Simulation data suggested that the DSED setup takes approximately 13.7 s longer than standard defibrillation [10]. While this may appear modest, such delays during cardiac arrest could meaningfully affect outcomes. More importantly, these findings highlight the absence of structured training pathways for DSED use. Currently, major resuscitation courses, such as the ERC Advanced Life Support (ALS), do not include DSED training, leaving providers without standardized guidance. This training gap raises important safety concerns, as providers may attempt DSED without adequate preparation, increasing the risk of errors or delays in critical phases of care. Given the current lack of standardized instruction, future research should explore whether integrating DSED-specific training into resuscitation curricula could enhance provider preparedness and improve safety.

The inter-shock interval is another critical factor. Evidence from one study [11] suggested that shorter intervals (<75 ms) were associated with significantly higher rates of VF termination. However, achieving such near-simultaneous shocks in practice requires precise coordination, which is challenging in the chaotic environment of out-of-hospital cardiac arrest. Importantly, these findings are derived from a single investigation and therefore require replication before they can be generalized. Additional concerns about potential device damage during closely timed shocks have further limited widespread adoption [12,14]. Unreliable synchronization may not only reduce effectiveness but also introduce the risk of equipment malfunction, indirectly affecting patient safety during resuscitation. Future research should not only confirm the optimal timing but also assess the real-world feasibility of achieving it across diverse EMS systems.

Implementation of DSED often depends on the presence of advanced or specially trained providers [11,22]. Managing pad placement, selecting appropriate vectors, and coordinating two devices introduces considerable operational complexity, particularly in high-stress scenarios. Evidence indicates that in many systems, DSED is applied late in resuscitation or inconsistently, reflecting provider hesitation and lack of familiarity [9,20]. Such variability in execution, especially under pressure, can result in deviations from optimal protocols and jeopardize the safety and effectiveness of care. Future research should therefore go beyond assessing clinical endpoints and actively evaluate the impact of structured training programs, ensuring that providers are competent in performing DSED under real-world conditions.

Variation across EMS protocols is another barrier. Some agencies attempt DSED after three failed shocks, while others wait until after five or more [18,23]. Differences in pad placement strategies and energy delivery further undermine reproducibility. Additionally, in several systems, DSED requires online medical authorization, introducing delays at a critical time [21]. By contrast, VC can be performed with a single defibrillator, but while technically easier, it has demonstrated less consistent survival benefit [18,25]. Lack of protocol harmonization introduces not only operational inefficiencies but also potential safety risks, particularly when treatment decisions are delayed or improvised. These inconsistencies reflect the lack of standardized guidelines, and they limit the external validity of available evidence.

Previous systematic reviews have predominantly evaluated DSED and VC from a clinical perspective. One review [2] reported potential improvements in ROSC and survival with DSED but stressed the heterogeneity and methodological weaknesses of included studies. Another [3] found higher VF termination rates but no consistent survival benefit, while a more recent analysis [26] also judged the evidence encouraging yet inconclusive due to small sample sizes and observational designs. Our review aligns with these conclusions regarding clinical uncertainty but adds a novel perspective: we systematically identified the practical barriers and methodological limitations that restrict real-world adoption. In doing so, we also emphasize that operational feasibility and provider safety are central components of patient safety in cardiac arrest management. Unlike earlier reviews, which focused mainly on clinical endpoints, our findings highlight that equipment availability, training deficits, and protocol inconsistency are equally critical determinants of feasibility and may explain, in part, the variability in clinical outcomes across studies.

### 4.1. Limitations of This Review

This review has several limitations. First, the analysis was restricted to English-language studies, potentially excluding relevant data from other regions. Second, the included studies displayed significant heterogeneity in RVF definitions, intervention protocols, and outcome reporting, which precluded a meta-analysis. Third, most available evidence was derived from retrospective or observational designs, and thus, the findings remain vulnerable to selection bias and residual confounding. These design limitations also limit conclusions about patient safety outcomes, such as neurological recovery or harm related to delayed or incorrect DSED application. Finally, publication bias cannot be excluded, as case reports and small case series with negative outcomes may have been underrepresented in the published literature. In addition, only two RCTs were identified, and most studies had small sample sizes and considerable clinical heterogeneity. None of the included studies achieved the maximum NOS quality score.

### 4.2. Future Research Directions

Future research should prioritize large, multicenter RCTs with standardized definitions of RVF and harmonized outcome measures, particularly including neurological function and long-term survival. Studies evaluating the integration of DSED and VC into resuscitation training curricula are also warranted, as current certification programs do not address these techniques. Furthermore, investigations should assess the cost-effectiveness and feasibility of these approaches in resource-limited settings, where the availability of defibrillators and consumables remains a major barrier. Special attention should be given to safety endpoints, such as adverse events, delays, or protocol deviations, to ensure patient-centered evaluation of these interventions. Finally, pragmatic implementation studies are needed to clarify optimal protocols for timing, pad placement, and shock sequencing, ensuring that clinical adoption is both evidence-based and operationally feasible.

## 5. Conclusions

This systematic review demonstrates that while DSED and VC hold promise for improving outcomes in refractory ventricular fibrillation, their clinical adoption is constrained by significant practical barriers and methodological limitations in the evidence base. Key obstacles include the requirement for additional equipment, delays in setup, lack of structured training, and variability across protocols, all of which limit feasibility in both prehospital and in-hospital settings. Moreover, the predominance of small, retrospective, and heterogeneous studies creates uncertainty regarding the true effectiveness of these techniques. Beyond clinical efficacy, our findings emphasize that safety-related challenges such as equipment readiness, staff competence, and procedural consistency must be addressed before these interventions can be safely integrated into routine care. Unlike prior reviews, this analysis highlights that practical barriers are as critical as clinical endpoints in determining real-world applicability. Addressing these challenges will require not only well-powered RCTs but also integration of training, standardization of protocols, and evaluation of cost-effectiveness. Until such evidence is available, the role of DSED and VC in standard resuscitation practice should be considered promising yet unproven.

## Figures and Tables

**Table 1:** Study Characteristics.

Author (Year)	Country	Study Design	Sample/Intervention	Outcomes	Clinical Barriers/Limitations
Eraniyan et al. (2025) [13]	USA	Retrospective case series	29 OHCA patients with RVF, DSED used	ROSC 24%, Survival 21%, Neurological outcome NR	Delays due to equipment/setup; small sample; registry design
Rahimi et al. (2024) [14]	Canada	Retrospective cohort	106 OHCA, DSED intervals analyzed	ROSC 24% (<75 ms); no survival/neurological difference	Timing precision critical; incomplete data; low power
Nordviste et al. (2024) [10]	Norway	Observational simulation	108 procedures by EMS teams	DSED delay ~13.7s vs. standard; feasible in simulation	Simulation only; no CPR/shocks; generalizability limited
Cheskes et al. (2024) [25]	Canada	Secondary analysis of RCT	345 OHCA, DSED/VC vs. standard	DSED: ROSC/survival benefit; VC improved VF termination	Small subgroup sizes; no post-ROSC care data
Verkaik et al. (2024) [15]	Netherlands	Observational registry	436 OHCA with ≥3 shocks	True RVF 5%; VF terminated in 95%	Hard to differentiate VF types in real-time; outcome scope limited
Narducci & Pedicino (2023) [18]	Italy	Cluster-RCT	405 OHCA with RVF	DSED: survival 30%, ROSC 46%, neuro 27%	COVID-related early stop; no long-term outcomes
Kim et al. (2020) [16]	South Korea	Retrospective pilot	38 IHCA with RVF/VT	DSiD better early outcomes; neuro not significant	Small sample; ED setting; coordination issues
Cheskes et al. (2020) [11]	Canada	Pilot cluster-RCT	152 OHCA, DSED/VC vs. standard	Feasibility 89.5%, ROSC improved	Pilot size; generalizability concerns; no power for outcomes
Mapp et al. (2019) [19]	USA	Matched case–control	205 OHCA (64 survivors matched)	No survival/neuro difference between DSD and standard	Late DSD use; small subgroups; variable CPR quality
Beck et al. (2019) [20]	USA	Retrospective cohort	310 OHCA with RVF	Lower ROSC/survival in DSD vs. standard	Selection bias; non-standardized DSD application
Cheskes et al. (2019) [21]	Canada	Retrospective cohort	252 OHCA, DSED vs. standard	Better early ROSC with DSED; NS overall	Online approval delays; observational design
Emmerson et al. (2017) [22]	UK	Retrospective observational	220 OHCA (45 DSED)	ROSC 38%, survival 7% with DSED	Late DSED, AP-only staff; small group
Ross et al. (2016) [23]	USA	Retrospective cohort	279 OHCA with RVF	NS differences in ROSC/survival/neuro	Inconsistent timing; missing data; selection bias
Cortez et al. (2016) [9]	USA	Retrospective case series	12 OHCA with RVF ≥5 shocks	ROSC 25%, neuro intact 17%	Delays in DSED; small, non-comparative study
Merlin et al. (2016) [24]	USA	Retrospective case series	7 OHCA with ≥3 shocks	Survival 43%, neuro intact 43%	Tiny sample; protocol adherence concerns
Cabañas et al. (2015) [1]	USA	Retrospective case series	10 OHCA with ≥5 shocks	ROSC 30%, no discharge survival	Very small group; no neuro/post-arrest data

**Table 2:** Newcastle–Ottawa Scale quality assessment of observational studies.

Study	Selection				Comparability	Outcomes			Total
Study	Representativeness	Selection (Non-Exposed)	Ascertainment	Outcome Not Present	Comparability	Assessment	Follow-Up Length	Follow-Up Adequacy	Total
Cheskes et al. (2024) [25]	*	*	*	*	**	*		*	**8**
Rahimi et al. (2024) [14]	*		*	*	*	*	*	*	**7**
Verkaik et al. (2024) [15]	*		*	*	*	*	*		**6**
Nordviste et al. (2024) [10]	*		*	*	*	*	*	*	**7**
Kim et al. (2020) [16]	*		*	*		*	*	*	**6**
Mapp et al. (2019) [19]	*	*	*	*	**	*		*	**8**
Beck et al. (2019) [20]	*		*	*	*	*	*	*	**7**
Cheskes et al. (2019) [21]	*		*	*	*	*	*	*	**7**
Emmerson et al. (2017) [22]	*		*	*	*	*	*	*	**7**
Ross et al. (2016) [23]	*		*	*	*	*	*	*	**7**

Stars indicate awarded points for each NOS domain. Selection (max 4), Comparability (max 2), and Outcome (max 3), with a maximum total score of 9.

**Table 3:** Methodological Quality Assessment of the Included Case Series Using the JBI Critical Appraisal Checklist (Q1–Q10).

Study	Q1	Q2	Q3	Q4	Q5	Q6	Q7	Q8	Q9	Q10
Eraniyan et al. 2025 [13]	Yes	Yes	Yes	Yes	Yes	No	Unclear	Yes	Yes	No
Merlin et al. 2016 [24]	Yes	Yes	Yes	Yes	Yes	Yes	Yes	Yes	Yes	Yes
Cortez et al. 2016 [9]	Yes	Yes	Yes	Yes	Yes	Yes	Yes	Yes	Yes	No
Cabañas et al. 2015 [1]	Yes	Yes	Yes	Yes	Yes	Yes	Yes	Yes	Yes	No

## Data Availability

No new data were created or analyzed in this study. Data sharing is not applicable to this article.

## Supplementary Materials

The following supporting information can be downloaded at: https://www.mdpi.com/article/10.3390/healthcare13202645/s1, File S1: PRISMA 2020 checklist; File S2: Joanna Briggs Institute (JBI) Critical Appraisal Checklist.

## Abbreviations

The following abbreviations are used in this manuscript:

VF	Ventricular Fibrillation
RVF	Refractory Ventricular Fibrillation
ERC	European Resuscitation Council
AHA	American Heart Association
RCT	Randomized Controlled Trial
DSED	Double Sequential External Defibrillation
VC	Vector Change
OHCA	Out-of-Hospital Cardiac Arrest
IHCA	In-Hospital Cardiac Arrest
CPR	Cardiopulmonary Resuscitation
ROSC	Return of Spontaneous Circulation
PRISMA	Preferred Reporting Items for Systematic Reviews and Meta-Analyses
NOS	Newcastle–Ottawa Scale
RoB 2	Cochrane Risk of Bias 2 Tool
EMS	Emergency Medical Services
