# Supplementary material for: Enhancing Patient Safety in Refractory Ventricular Fibrillation: A Systematic Review of Double Sequential and Vector Change Defibrillation Barriers
Source: Healthcare (Basel). 2025 Oct 21;13(20):2645. doi: 10.3390/healthcare13202645 (PMC12563664; doi:10.3390/healthcare13202645)
Supplement: Supplementary file 1 [file healthcare-13-02645-s001.zip › healthcare-3898629-file S2.pdf]

Question	Yes	No	Unclear	Not applicable
1.Were there clear criteria for inclusion in the case series?				
2.Was the condition measured in a standard, reliable way for all participants included in the case series?				
3.Were valid methods used for identification of the condition for all participants included in the case series?				
4.Did the case series have consecutive inclusion of participants?				
5.Did the case series have complete inclusion of participants?				
6.Was there clear reporting of the demographics of the participants in the study?				
7.Was there clear reporting of clinical information of the participants?				
8.Were the outcomes or follow up results of cases clearly reported?				
9.Was there clear reporting of the presenting site(s)/clinic(s) demographic information?				
10. Was statistical analysis appropriate?				
